# Analysis of the attitudes of people with type 2 diabetes mellitus in Primary Health Care

**DOI:** 10.1590/1980-220X-REEUSP-2025-0549en

**Published:** 2026-10-12

**Authors:** Amilton Diniz dos Santos, Ravena Melo e Silva, Maria Rita Sousa da Silva, Irisdalva França Soares Brito, Maria Edileuza Soares Moura

**Affiliations:** 1 Universidade Estadual do Maranhão, Programa de Pós-graduação em Biodiversidade, Ambiente e Saúde, Caxias, MA, Brazil.; 2 Universidade Estadual do Maranhão, Departamento de Ciências da Saúde, Caxias, MA, Brazil.; 3 Universidade Estadual do Maranhão, Programa de Residência Multiprofissional em Saúde da Família, Caxias, MA, Brazil.

**Keywords:** Attitude, Primary Health Care, Self Care, Health Education, Diabetes Mellitus, Type 2

## Abstract

**Objective:**

To analyze the attitudes of people with type 2 diabetes mellitus toward the disease.

**Method:**

This cross-sectional study was conducted in Primary Health Care with 129 participants diagnosed with type 2 diabetes mellitus. The Diabetes Attitude Questionnaire (ATT-19) was administered. Statistical analysis included logistic regression, assessment of multicollinearity, model fit assessment, and statistical significance testing.

**Results:**

Participants were predominantly female (n = 84; 65.12%), self-identified as mixed-race (n = 63; 48.84%), retired (n = 73; 56.59%), had completed elementary school (n = 49; 37.98%), and were married or living in a stable union (n = 64; 49.61%). Male sex showed a trend toward an association with a negative attitude (OR = 2.66; 95%CI: 0.97–8.10; p = 0.067), whereas living alone demonstrated a protective effect (OR = 0.10; 95%CI: 0.01–0.73; p = 0.032).

**Conclusion:**

A predominance of negative attitudes toward type 2 diabetes mellitus, as measured by the ATT-19, was identified. A more negative attitude was associated with male sex, whereas living alone had a protective effect, indicating the need for strategies focused on attitudes in Primary Health Care.

## INTRODUCTION

Noncommunicable diseases (NCDs) comprise a group of diseases with a prolonged course, generally characterized by an insidious onset and slow progression. They are characterized by persistence over time and the need for continuous care aimed at symptom control, reduction of complications, and maintenance of functionality^(1)^. The main NCDs include cardiovascular diseases, cancer, chronic respiratory diseases, and diabetes mellitus (DM). These conditions affect the quality of life of those living with them, interfere with their autonomy and performance of daily activities, and require regular follow-up by healthcare professionals^(2)^.

Among NCDs, type 2 diabetes mellitus (T2DM) is one of the most prevalent chronic conditions and one of the conditions with the greatest impact on healthcare systems. It is characterized by persistent hyperglycemia resulting from alterations in insulin production or action^(3,4,5,6)^. Globally, T2DM accounts for more than 90% of all diabetes cases, and its prevalence has increased across several countries, driven by demographic changes, urbanization, and greater exposure to behavioral risk factors^(7)^. In Brazil, a similar trend has been observed, with a marked increase in the number of people living with the disease over recent decades, reinforcing the need for care strategies focused on disease management^(8)^.

Against this background, addressing T2DM is aligned with Target 3.4 of Sustainable Development Goal 3 (Good Health and Well-being), which aims to reduce premature mortality from NCDs by 30% by 2030. This target encompasses effective health system interventions, including prevention, risk control, appropriate treatment, and access to essential healthcare services^(9)^.

In this context, T2DM management requires regular clinical follow-up and the active participation of individuals in managing their disease. The adoption of self-care practices, such as adherence to dietary and medication plans, regular physical activity, and monitoring of signs and symptoms, is essential for achieving metabolic balance and reducing complications^(10)^. However, the effectiveness of these actions is directly related to treatment adherence, which is influenced by several factors, particularly the attitudes and beliefs individuals hold regarding their health condition, as these are determinants of maintaining healthy behaviors over time^(11)^.

Attitude can be understood as a cognitive disposition that guides decision-making in response to the demands of treatment and coping with the disease. It is a subjective component that reflects how individuals perceive, accept, and position themselves regarding their own health condition^(12)^. When positive, this attitude promotes engagement in self-care practices and adherence to treatment, contributing to better clinical outcomes and a greater sense of well-being. Such attitudes are strongly influenced by the knowledge individuals acquire over time, whether through personal experiences or professional guidance, both of which provide the basis for disease management^(13,14,15)^.

Despite the relevance of this construct, studies investigating the attitudes of people with T2DM in the context of Primary Health Care (PHC) remain limited, particularly in northeastern Brazil. Most studies focus on clinical indicators, with less attention given to dimensions related to attitudes toward coping with the disease.

Therefore, investigating the attitudes of people with T2DM receiving care in PHC may contribute to a better understanding of how these individuals perceive and position themselves regarding the disease within their healthcare context. This knowledge may provide support for planning healthcare actions aligned with users’ experiences and perceptions. Accordingly, the present study aimed to analyze the attitudes of people with T2DM toward the disease.

## METHOD

### Study Design

This was a cross-sectional study with a quantitative and exploratory design conducted in the municipality of Caxias, Maranhão, Brazil. The study design and reporting followed the STrengthening the Reporting of OBservational studies in Epidemiology checklist recommendations for cross-sectional studies.

### Study Setting

The study population consisted of individuals served by a PHC team in the municipality’s urban area. The selection of this Basic Health Unit is justified by its status as a model unit and its role as a training site for professionals in *Universidade Estadual do Maranhão*’s Multiprofessional Residency Program in Family Health. Furthermore, its extensive catchment area and the significant number of users diagnosed with T2DM make it a promising setting for this study.

### Population and Selection Criteria

The PHC team provided follow-up care to 198 individuals diagnosed with T2DM. Eligible participants were individuals with a clinical diagnosis of T2DM, aged 18 years or older, registered with and followed by the selected PHC team, and with preserved cognitive capacity to respond to the data collection instrument, even if assistance from a family member, caregiver, or companion was required. Individuals with impairments that hindered verbal communication and/or understanding of the instrument were excluded.

A convenience sample was used based on participants’ accessibility and availability during the data collection period, which may have introduced selection bias and limited the representativeness of the population followed by the PHC team as well as the generalizability of the findings. The diagnosis of T2DM was confirmed through electronic medical records at the health unit, considering clinical diagnoses previously established by a physician during PHC follow-up.

### Sample Size Calculation

The sample size calculation was performed using the formula for finite populations: n = N·Z^2^·p·(1–p)1/(N-1)·e^2^ + Z^2^·p·(1–p)^(16)^. A heterogeneous population, a 95% confidence level (Z = 1.96), a 5% margin of error (e = 0.05), and an estimated prevalence of 50% (p = 0.5) were considered, resulting in a sample of 131 participants. During data collection, two participants were excluded because they had impairments that hindered verbal communication and understanding of the instrument, resulting in a final sample of 129 participants.

### Data Collection

Data were collected between March and June 2025 at the health unit and in participants’ homes, according to their availability. Community health workers, who were responsible for participant follow-up, assisted with data collection. The questionnaires were administered individually in a private setting to ensure privacy, avoid embarrassment, and guarantee the confidentiality of the information. The mean interview duration was approximately 25 minutes (SD = 5), ranging from 20 to 40 minutes.

A structured questionnaire developed by the researchers was used to obtain information on sociodemographic variables (age, sex, educational level, marital status, occupation, income, and household composition) and clinical variables (time since diagnosis and chronic complications). Age was analyzed as a continuous variable and expressed as mean, standard deviation, and variance. Time since diagnosis was categorized into four groups: ≤1 year, 1–5 years, 6–10 years, and >10 years.

To assess participants’ attitudes toward T2DM, the Diabetes Attitude Questionnaire (ATT-19), translated into Portuguese and validated in Brazil, was used^(17)^. This instrument assesses psychological adjustment to DM and was developed in response to the need to assess psychological and emotional aspects related to the disease. It consists of 19 items grouped into six factors: (a) stress associated with DM; (b) receptivity to treatment; (c) confidence in treatment; (d) personal efficacy; (e) health perception; and (f) social acceptance. Items 11, 15, and 18 are reverse scored. Responses are measured using a five-point Likert scale ranging from strongly disagree (score 1) to strongly agree (score 5). The total score ranges from 19 to 95 points. A score lower than 70 indicates a negative attitude toward the disease^(17)^.

The cut-off point of <70 was adopted according to the recommendation of the Brazilian validation study of the questionnaire, which established this value as indicative of a negative attitude. Additionally, a complementary analysis of the total ATT-19 score as a continuous variable was performed to explore possible associations without loss of information resulting from dichotomization.

### Data Analysis and Processing

Statistical analyses were performed using RStudio (version 2025.05.0), with the dplyr package for data manipulation and organization. Continuous variables were described using measures of central tendency and dispersion, including mean, standard deviation, and variance. Categorical variables were described using absolute and relative frequencies to characterize the distribution of the data.

To investigate factors associated with a negative attitude toward T2DM, logistic regression models were fitted. Initially, a simple logistic regression model was performed for each sociodemographic variable to identify potential predictors for the multiple model. Subsequently, all variables were included in an initial model, and multicollinearity was assessed using the Variance Inflation Factor, with values >5 considered indicative of high collinearity.

Variables were retained in the model based on their theoretical and epidemiological relevance, and their exclusion was jointly assessed according to model fit criteria (Akaike Information Criterion [AIC]) and collinearity. Model 1 corresponds to the adjusted model including all variables selected based on theoretical and statistical criteria. Model 2 refers to the final model obtained after the joint assessment of statistical significance, collinearity, and goodness of fit.

For each variable, Odds Ratios (ORs) with their respective 95% Confidence Intervals (95%CIs) and p-values were estimated, adopting a significance level of p <0.05. The likelihood ratio test (LRT) was used to assess the inclusion of predictor variables in the multiple model compared with the nested model without predictors using the lmtest package.

The LRT was also used to compare the nested models, assessing whether the joint inclusion of the variables significantly improved model fit compared with the reduced model. In addition, model comparison considered the AIC, with the model presenting the lowest AIC regarded as the most appropriate, indicating the best balance between model fit and parsimony. Furthermore, the Hosmer-Lemeshow test (HLT), using the ResourceSelection package, was applied to assess model adequacy by examining the agreement between predicted and observed probabilities.

### Ethical Aspects

The research project was approved by the Research Ethics Committee of *Universidade Estadual do Maranhão* under Opinion 5,967,691 and Certificate of Presentation for Ethical Consideration 67497823.0.0000.5554. All participants were previously informed about the objectives and methods of the study and, after agreeing to participate, signed the Informed Consent Form.

## RESULTS

The sample consisted of 129 participants with a mean age of 64.39 ± 11.89 years, indicating a predominance of older adults. Females were more prevalent, accounting for 84 (65.12%) participants. Most participants self-identified as mixed-race, totaling 63 (48.84%) individuals. Regarding marital status, 64 (49.61%) were married or living in a stable union. Elementary school education was the most frequent educational level, reported by 49 (37.98%) participants. Retirement was the most common occupation, reported by 73 (56.59%) participants. Most participants had an income of up to one minimum wage (79; 61.24%) and lived with family members (52; 40.31%) (Table 1).

**Table 1:** Sociodemographic profile of participants and attitudes toward the disease according to the Diabetes Attitude Questionnaire – Caxias, MA, Brazil, 2025.

	Negative attitude (n = 92)	Positive attitude (n = 37)	Total N = 129
Age			
Mean	63.5	66.6	64.39
Standard deviation	11	13.7	11.89
Variance	122	187.7	141.55
	**n (%)**	**n (%)**	**n (%)**
Sex			
Female	57 (61.96)	27 (72.97)	84 (65.12)
Male	35 (38.04)	10 (27.03)	45 (34.88)
Ethnicity			
Mixed-race	51 (55.43)	12 (32.43)	63 (48.84)
Black	25 (27.17)	17 (45.95)	42 (32.56)
White	16 (17.39)	8 (21.62)	24 (18.60)
Marital status			
Married/stable union	46 (50.00)	18 (48.65)	64 (49.61)
Single	27 (29.35)	10 (27.03)	37 (28.68)
Widowed	17 (18.48)	8 (21.62)	25 (19.38)
Divorced	2 (2.17)	1 (2.70)	3 (2.33)
Educational level			
Literacy	25 (27.17)	8 (21.62)	33 (25.58)
Elementary school	38 (41.30)	11 (29.73)	49 (37.98)
High school	12 (13.04)	6 (16.22)	18 (13.95)
Higher education/specialization	5 (5.43)	4 (10.81)	9 (6.98)
No formal education	12 (13.04)	8 (21.62)	20 (15.50)
Occupation			
Retired	52 (56.52)	21 (56.76)	73 (56.59)
Self-employed	31 (33.70)	14 (37.84)	45 (34.88)
Salaried worker	5 (5.43)	1 (2.70)	6 (4.65)
Other	4 (4.35)	1 (2.70)	5 (3.88)
Income			
Up to one minimum wage	57 (61.96)	22 (59.46)	79 (61.24)
More than one minimum wage	6 (6.52)	3 (8.11)	9 (6.98)
No income	29 (31.52)	12 (32.43)	41 (31.78)
Household composition			
Living only with spouse	15 (16.30)	2 (5.41)	17 (13.18)
Living only with family members	36 (39.13)	16 (43.24)	52 (40.31)
Living with spouse and family members	26 (28.26)	11 (29.73)	37 (28.68)
Living alone	15 (16.30)	8 (21.62)	23 (17.83)

Source: the authors, 2025.

Table 1 also shows the scores obtained from the assessment of participants’ attitudes toward T2DM using the ATT-19. Most participants scored below 70 points, indicating a negative attitude toward the disease, totaling 92 (71.32%) individuals.

Regarding clinical characteristics, 42 (32.56%) participants had been living with a diagnosis of T2DM for six to ten years. Regarding disease-related complications, the absence of complications predominated, totaling 101 (78.29%) participants. Among those with complications, retinopathy was the most prevalent, affecting 19 (14.73%) individuals (Table 2). Information on clinical complications was obtained from the patients’ electronic medical records at the health unit.

**Table 2:** Clinical profile of participants according to attitudes toward the disease based on the Diabetes Attitude Questionnaire – Caxias, MA, Brazil, 2025.

	Negative attitude (n = 92)	Positive attitude (n = 37)	Total N = 129
	n (%)	n (%)	n (%)
Time since diagnosis (in years)			
< 1	8 (8.70)	2 (5.41)	10 (7.75)
1 to 5	27 (29.35)	12 (32.43)	39 (30.23)
6 to 10	30 (32.61)	12 (32.43)	42 (32.56)
> 10	27 (29.35)	11 (29.73)	38 (29.46)
Complications			
None	74 (80.43)	27 (72.97)	101 (78.29)
Retinopathy	12 (13.04)	7 (18.92)	19 (14.73)
Renal	2 (2.17)	2 (5.41)	4 (3.10)
Vascular	2 (2.17)	1 (2.70)	3 (2.33)
Neuropathy	2 (2.17)	0 (0.00)	2 (1.55)

Source: the authors, 2025.

The association tests (chi-square and Fisher’s exact test) between sociodemographic variables, clinical variables, and attitude (negative/positive) showed no statistically significant associations (p < 0.05). Therefore, a logistic regression analysis was performed to identify factors potentially associated with a negative attitude toward T2DM (Table 3).

**Table 3:** Multiple logistic regression: factors associated with negative attitudes toward the disease among individuals diagnosed with type 2 diabetes mellitus according to the Diabetes Attitude Questionnaire – Caxias, MA, Brazil, 2025.

Variables included in the model	Model 1	Model 2
	OR (95%CI) p-value	OR (95%CI) p-value
Age	0.95 (0.89; 1) 0.053	0.96 (0.92; 1.01) 0.095
Sex (Ref = female)		
Male	2.96 (1.05; 9.27) 0.049*	2.66 (0.97; 8) 0.067
Ethnicity (Ref = white)		
Mixed-race	2.36 (0.69; 8.04) 0.166	2.36 (0.7; 8) 0.162
Black	0.67 (0.18; 2.30) 0.530	0.66 (0.18; 2.23) 0.51
Marital status (Ref = married/stable union)		
Divorced	2.37(0.1; 82.79) 0.592	2.7 (0.13; 87.29) 0.52
Single	2.46 (0.57; 11.05) 0.229	2.76 (0.66; 12.08) 0.166
Widowed	3.58 (0.67;19.72) 0.136	4.33 (0.86; 22.94) 0.078
Educational level (Ref = literacy)		
Elementary school	0.88 (0.23; 3.31) 0.852	0.89 (0.24; 3.29) 0.867
High school	0.38 (0.07; 1.87) 0.230	0.36 (0.07; 1.73) 0.201
Higher education/specialization	0.17 (0.02; 1.27) 0.085	0.18 (0.03; 1.07) 0.059
Without education	0.58 (0.15; 2.24) 0.429	0.54 (0.14; 2.06) 0.367
Household composition (Ref = living only with spouse)		
Living only with family members	0.16 (0.02; 1.17) 0.088	0.15 (0.02; 1.03) 0.07
Living with spouse and family members	0.29 (0.03; 1.56) 0.22	0.31 (0.04; 1.65) 0.202
Living alone	0.11 (0.01; 0.80) 0.040*	0.1 (0.01; 0.73) 0.032*
Income (Ref = up to one minimum wage)		
More than one minimum wage	0.72 (0.11; 5.59) 0.733	–
No income	0.50 (0.13; 1.75) 0.279	–
**Model assessment criteria**		
AIC	169.32	164.12
VIF	<5	<5
LRT (p-value)	0.1553	0.1153
HLT (p-value)	0.213	0.589

Legend: Ref – reference category;

*Statistically significant p-value at the 95% confidence level; AIC – Akaike Information Criterion; VIF – Variance Inflation Factor; LRT – Likelihood Ratio Test; HLT – Hosmer-Lemeshow Test; 95%CI – 95% confidence interval.

Source: the authors, 2025.

In model 1, male sex was significantly associated with a negative attitude (OR = 2.96; 95%CI: 1.05–9.27; p = 0.049), indicating that men were more likely to exhibit a negative attitude toward the disease than women. In addition, living alone showed a protective effect (OR = 0.11; 95%CI: 0.01–0.80; p = 0.040), indicating that, compared with the reference group (living only with a spouse), individuals living alone were less likely to have a negative attitude.

In model 2, after adjustment, living alone remained a protective factor (OR = 0.10; 95%CI: 0.01–0.73; p = 0.032). Male sex showed only a trend toward an association with a negative attitude (OR = 2.66; 95%CI: 0.97–8.10; p = 0.067), without reaching statistical significance at the 5% level. No other variable showed a statistically significant association in the final models. The models demonstrated adequate fit according to the HLT (p = 0.213 for model 1 and p = 0.589 for Model 2), in addition to satisfactory AIC values (169.32 and 164.12, respectively) (Table 3).

## DISCUSSION

The use of the ATT-19 identified that 71.32% of the participants had negative attitudes toward T2DM. In the multivariable analyses, male sex was associated with a higher likelihood of negative attitudes (OR = 2.96; 95%CI: 1.05–9.27; p = 0.049), whereas living alone showed a protective effect (OR = 0.11; 95%CI: 0.01–0.80; p = 0.040). Factors such as mixed-race ethnicity, low educational level, a monthly income of up to one minimum wage, and a time since diagnosis of six to ten years characterized the study sample and were described as variables without a statistically significant association.

The use of validated instruments represents an essential strategy in healthcare practice, as it enables the standardization of language among healthcare professionals, thereby facilitating interdisciplinary communication^(18)^. In addition, it serves as a tool for assessing therapeutic responses, comparing data over time, and understanding aspects related to the management of chronic conditions such as T2DM, allowing a more consistent analysis of behaviors and attitudes toward the disease^(19)^.

The variable “age” showed a borderline association with negative attitudes toward T2DM diagnosis, as observed in model 1 (OR = 0.95; 95%CI: 0.89–1.00; p = 0.053). Although the p-value was slightly above the conventional threshold for statistical significance (p < 0.05), the finding suggests a trend indicating that increasing age may be associated with a lower likelihood of developing a negative attitude toward the disease.

This finding may be interpreted in light of psychosocial factors and experiences related to living with a chronic condition. Since older adults are more frequently exposed to experiences associated with chronic illness, they may develop more effective adaptive strategies, greater resilience, and a more realistic acceptance of living with T2DM. They may also have a more mature perception of the limitations imposed by the condition^(20)^.

Females predominated in the study sample, a finding similar to that reported in another study^(21)^, which included 936 individuals with T2DM and found that 63.4% were women. This predominance may be related to more frequent self-care behaviors among women, greater concern for their health, and regular use of preventive and follow-up healthcare services^(22)^. Furthermore, studies suggest that women tend to adhere more closely to T2DM care and treatment, which may explain the higher detection rate of the disease in this group^(20,21)^.

In the present study, male participants had higher negative attitude scores toward T2DM, with a statistically significant association in model 1. However, because this association did not remain statistically significant in model 2 (p = 0.067), this finding should be interpreted as a trend. This result is consistent with evidence indicating lower engagement of men in preventive practices and lower adherence to treatment for chronic diseases compared with women^(23)^.

Most participants self-identified as mixed-race, which may be related to the ethnic characteristics of the Brazilian population and the region where the study was conducted. Recent studies have reported similar findings, highlighting the increasing use of ethnic-racial self-identification in health surveys, allowing individuals to identify themselves according to their own perception^(24,25)^.

This phenomenon may be associated with the strengthening of public policies aimed at promoting equity in healthcare. Understanding the ethnic-racial distribution among people with T2DM is essential because socioeconomic and cultural factors, as well as potential inequalities in access to healthcare services, may affect disease management and influence attitudes toward treatment.

Low educational attainment was predominant in this study and may reflect historical inequalities in access to education in Brazil until the twentieth century. Similar findings have been reported in the literature, including a study that assessed DM-related knowledge, attitudes toward self-care, and associated factors, indicating that educational level may be associated with lower knowledge about the disease^(26)^. In this regard, the literature indicates that this condition may be associated with difficulties in understanding and following therapeutic recommendations, which may increase health risks.

Similarly, a recent study identified a significant association between low educational level and an increased risk of T2DM. The authors emphasized that educational attainment is an important social determinant of health because it may influence access to information, healthcare services, and self-care practices related to disease prevention and management^(27)^.

Retired participants with a monthly income of up to one minimum wage predominated in the sample, a finding consistent with previous studies^(21,26)^. Low income is a matter of concern because it may represent a barrier to meeting individuals’ basic needs and may hinder continuous access to the resources required for DM management. Limited household income remains a critical factor in the context of T2DM, as unstable living conditions, the prolonged duration of treatment, and healthcare-related costs may pose challenges to disease management. Furthermore, financial constraints may hinder the adoption and maintenance of healthy eating habits, which are a fundamental component of glycemic control. In this context, the literature indicates that unfavorable socioeconomic conditions may limit access to the resources required for treatment, negatively affecting disease management and individuals’ quality of life^(28)^.

Regarding marital status, a higher proportion of participants were married or living in a stable union. Similar findings were reported in a study that analyzed the correlation between depressive symptoms, attitudes, and self-care among older adults with T2DM, involving 144 participants receiving care in PHC in João Pessoa^(29)^. Marital status has been described in the literature as a factor related to family dynamics and self-care, as household composition may be associated with treatment adherence^(30)^.

Despite the positive influence generally attributed to family support, the multivariable analysis of this study showed that living alone was associated with a protective effect against negative attitudes toward T2DM (OR = 0.11; 95%CI: 0.01–0.80; p = 0.040). Considering the small number of participants who lived alone and the wide confidence intervals, this finding should be interpreted with caution, as the instability of the estimate or the influence of unmeasured factors cannot be ruled out^(28)^.

In the present study, most participants had been living with a diagnosis of T2DM for six to ten years, a characteristic that reinforces the chronic and progressive nature of the disease. Furthermore, 78.29% had no DM-related complications, whereas among those with complications, retinopathy was the most prevalent (14.73%). The literature indicates that a longer duration of the disease is directly associated with an increased risk of microvascular and macrovascular complications because of the cumulative effects of poor glycemic control and insulin resistance over time^(3,5)^. These complications, particularly those affecting vision and the lower extremities, are associated with reduced functional capacity and poorer quality of life, making adherence to self-care more difficult^(5,6)^.

Among the study limitations, the cross-sectional design precludes establishing temporal or causal relationships between the variables. The use of a convenience sample may have introduced selection bias, thereby limiting the generalizability of the findings. The small sample size in some categories may also have reduced the statistical power. Although information on complications was obtained from medical records, other data relied on participants’ self-reports, which may have been influenced by personal interpretations and the tendency to provide socially desirable responses.

Additionally, the absence of objective measures of glycemic control, such as glycated hemoglobin, limited a more accurate assessment of participants’ clinical condition. The lack of detailed information regarding treatment, including medication use, treatment adherence, and duration of follow-up, also restricted the understanding of factors that may influence the observed attitudes. Furthermore, the possibility of residual confounding resulting from variables that were not measured or controlled for in the analytical model cannot be excluded.

Moreover, the sociodemographic profile of the sample, which consisted predominantly of women and individuals with low educational attainment, may not reflect the diversity of people living with T2DM. These factors should be considered when interpreting the findings and highlight the need for future studies involving more diverse samples to expand the understanding of attitudes across different social contexts.

## CONCLUSION

The findings of this study highlight the complexity of attitudes toward the diagnosis and treatment of T2DM among individuals living with the disease. A high frequency of negative attitudes toward T2DM was observed among participants assessed using the ATT-19. Among the variables analyzed, male sex was associated with a greater likelihood of negative attitudes, whereas living alone was associated with a lower likelihood of negative attitudes. These findings contribute to a better understanding of DM-related attitudes among individuals receiving care in PHC.

## Data Availability

The entire dataset supporting the results of this study was published in the article itself.
