# Supplementary material for: Analysis of the attitudes of people with type 2 diabetes mellitus in Primary Health Care
Source: Rev Esc Enferm USP. 2026 Oct 12;60:e20250549. doi: 10.1590/1980-220X-REEUSP-2025-0549en (PMC13675011; doi:10.1590/1980-220X-REEUSP-2025-0549en)
Supplement: Translation

# Translation: Análise das atitudes de pessoas com diabetes mellitus tipo 2 na Atenção Primária à Saúde

**DOI:** 10.1590/1980-220X-REEUSP-2025-0549pt

**Authors:** Amilton Diniz dos Santos, Ravena Melo e Silva, Maria Rita Sousa da Silva, Irisdalva França Soares Brito, Maria Edileuza Soares Moura

**Affiliations:** 1 Universidade Estadual do Maranhão, Programa de Pós-graduação em Biodiversidade, Ambiente e Saúde, Caxias, MA, Brasil.; 2 Universidade Estadual do Maranhão, Departamento de Ciências da Saúde, Caxias, MA, Brasil.; 3 Universidade Estadual do Maranhão, Programa de Residência Multiprofissional em Saúde da Família, Caxias, MA, Brasil.

**Keywords:** Atitude, Atenção Primária à Saúde, Autocuidado, Educação em Saúde, Diabetes Mellitus Tipo 2

## Abstract

**Objetivo:**

Analisar as atitudes de pessoas com diabetes mellitus tipo 2 em relação à doença.

**Método:**

Trata-se de um estudo transversal realizado na Atenção Primária à Saúde com 129 participantes diagnosticados com diabetes mellitus tipo 2. Aplicou-se o instrumento *Diabetes Attitude Questionnaire* (ATT-19). A análise estatística incluiu regressão logística, com verificação de multicolinearidade, ajuste do modelo e significância estatística.

**Resultados:**

Observou-se predominância do sexo feminino (n = 84; 65,12%), autodeclarados pardos (n = 63; 48,84%), aposentados (n = 73; 56,59%), com ensino fundamental (n = 49; 37,98%) e casados ou em união estável (n = 64; 49,61%). O sexo masculino apresentou tendência de associação com atitude negativa (OR = 2,66; IC95%: 0,97–8,10; p = 0,067), enquanto morar sozinho demonstrou efeito protetor (OR = 0,10; IC95%: 0,01–0,73; p = 0,032).

**Conclusão:**

Evidenciou-se a predominância de atitudes negativas diante do diabetes mellitus tipo 2, mensuradas pelo ATT-19, com maior atitude negativa associada ao sexo masculino e um efeito protetor para quem mora sozinho, indicando a necessidade de estratégias voltadas às atitudes na Atenção Primária à Saúde.

## INTRODUÇÃO

As doenças crônicas não transmissíveis (DCNTs) englobam um grupo de doenças de evolução prolongada, geralmente de início insidioso e progressão lenta. São caracterizadas pela persistência ao longo do tempo e pela necessidade de cuidados contínuos voltados ao controle de sintomas, à redução de complicações e à manutenção da funcionalidade^(1)^. Entre as principais DCNTs, destacam-se as doenças cardiovasculares, os cânceres, as doenças respiratórias crônicas e o diabetes mellitus (DM). Essas condições impactam a qualidade de vida das pessoas acometidas, interferem em sua autonomia e no desenvolvimento de atividades diárias e exigem acompanhamento regular pelos profissionais de saúde^(2)^.

Entre as DCNTs, o diabetes mellitus tipo 2 (DM2) é uma das condições crônicas mais prevalentes e de maior impacto para os sistemas de saúde, sendo caracterizada por hiperglicemia persistente decorrente de alterações na produção ou na ação da insulina^(3,6)^. Globalmente, o DM2 representa mais de 90% de todos os casos, e sua prevalência tem aumentado em diversos países, impulsionada por mudanças demográficas, urbanização e maior exposição a fatores de risco comportamentais^(7)^. No Brasil, observa-se uma tendência semelhante, com crescimento expressivo do número de pessoas vivendo com a doença nas últimas décadas, o que reforça a necessidade de estratégias de cuidado voltadas ao manejo da condição^(8)^.

Diante desse cenário, o enfrentamento do DM2 está alinhado à meta 3.4 do Objetivo do Desenvolvimento Sustentável 3 (Saúde e Bem-estar), que propõe reduzir em 30% a mortalidade prematura por DCNTs até 2030. Essa meta contempla intervenções eficazes no sistema de saúde, incluindo prevenção, controle de risco, tratamento adequado e acesso a cuidados essenciais^(9)^.

Nesse contexto, o controle do DM2 exige acompanhamento clínico periódico e participação ativa do próprio indivíduo no manejo da doença. A adoção de práticas de autocuidado, como a adesão ao plano alimentar e medicamentoso, prática de atividade física regular e monitoração dos sinais e sintomas, é fundamental para alcançar o equilíbrio metabólico e reduzir complicações^(10)^. No entanto, a efetividade dessas ações está diretamente relacionada ao grau de adesão ao tratamento, influenciado por diferentes fatores, destacando-se as atitudes e crenças que o indivíduo possui em relação à sua condição de saúde, os quais são determinantes para a manutenção de comportamentos saudáveis ao longo do tempo^(11)^.

A atitude pode ser compreendida como uma disposição cognitiva que orienta a tomada de decisão diante das exigências do tratamento e do enfrentamento da doença. Trata-se de um componente subjetivo que reflete a forma como o indivíduo percebe, aceita e se posiciona em relação à própria condição de saúde^(12)^. Quando positiva, essa atitude favorece o engajamento em práticas de autocuidado e a adesão ao tratamento, contribuindo para melhores resultados clínicos e sensação de bem-estar. Essa postura é fortemente influenciada pelo conhecimento que o indivíduo constrói ao longo do tempo, seja por meio de experiências pessoais, seja por meio de orientações profissionais, que fornecem subsídios para o seu manejo^(13,14,15)^.

Apesar da relevância desse construto, ainda são limitadas as investigações que analisam as atitudes de pessoas com DM2 no contexto da Atenção Primária à Saúde (APS), particularmente em regiões do Nordeste brasileiro. A maior parte da pesquisa se concentra em indicadores clínicos, havendo menor atenção às dimensões referentes às atitudes relacionadas ao enfrentamento da doença.

Nesse sentido, investigar as atitudes de pessoas com DM2 acompanhadas na APS pode contribuir para ampliar a compreensão sobre como esses indivíduos percebem e se posicionam diante da doença em seu contexto de cuidado. Esse conhecimento pode fornecer subsídios para o planejamento de ações de saúde alinhadas às experiências e percepções dos usuários. Dessa forma, o presente estudo teve como objetivo analisar as atitudes de pessoas com DM2 em relação à doença.

## MÉTODO

### Tipo do Estudo

Trata-se de um estudo transversal de abordagem quantitativa e caráter exploratório realizado no município de Caxias, localizado no estado do Maranhão. O delineamento e o relato da pesquisa seguiram as recomendações do *checklist STrengthening the Reporting of OBservational studies in Epidemiology*, direcionado para estudos transversais.

### Local do Estudo

Selecionou-se a população atendida por equipe de APS da zona urbana do município. A seleção da Unidade Básica de Saúde é justificada por sua caracterização como unidade modelo, além de ser campo de prática para profissionais vinculados ao Programa de Residência Multiprofissional em Saúde da Família da Universidade Estadual do Maranhão. Destacam-se, ainda, sua ampla área de abrangência e o expressivo número de usuários com diagnóstico de DM2, o que a torna um cenário promissor para o desenvolvimento do estudo.

### População e Critérios de Seleção

A equipe de APS acompanha 198 indivíduos com diagnóstico de DM2. Foram incluídos no estudo indivíduos com diagnóstico clínico de DM2, com idade igual ou superior a 18 anos, cadastrados e acompanhados pela equipe de APS selecionada e com capacidade cognitiva preservada para responder ao instrumento de coleta de dados, ainda que com auxílio de familiar, cuidador ou acompanhante. Foram excluídos indivíduos com comprometimentos que dificultassem a comunicação verbal e/ou compreensão do instrumento aplicado.

A amostra foi selecionada por conveniência, de acordo com a acessibilidade e a disponibilidade dos participantes no período da coleta dos dados, o que pode introduzir um viés de seleção e limitar a representatividade da população acompanhada pela equipe e a generalização dos achados. O diagnóstico de DM2 foi confirmado por meio de registro em prontuário eletrônico da unidade de saúde, considerando os diagnósticos clínicos previamente estabelecidos por um profissional médico, conforme acompanhamento na APS.

### Cálculo do Tamanho da Amostra

O cálculo amostral foi realizado utilizando a seguinte fórmula para populações finitas: n = N·Z^2^·p·(1–p)/(N–1)·e^2^+Z^2^·p·(1–p)^(16)^. Considerou-se uma população heterogênea, um nível de confiança de 95% (Z = 1,96), uma margem de erro de 5% (e = 0,05) e uma prevalência estimada de 50% (p = 0,5), resultando em uma amostra de 131 participantes. Durante a etapa de coleta de dados, dois participantes foram excluídos da pesquisa por apresentarem comprometimentos que dificultavam a comunicação verbal e a compreensão do instrumento aplicado, resultando em uma amostra final de 129 participantes.

### Coleta de Dados

A coleta de dados foi realizada entre março e junho de 2025 na unidade de saúde e no domicílio dos participantes, conforme a disponibilidade dos mesmos. Contou-se com o apoio de agentes comunitários de saúde, responsáveis pelo acompanhamento dos participantes. A aplicação dos questionários foi realizada de forma individual, em local reservado, visando garantir a privacidade, evitar constrangimentos e assegurar a confidencialidade das informações. O tempo médio de cada entrevista foi de aproximadamente 25 minutos (DP = 5), com tempo mínimo de 20 minutos e máximo de 40 minutos.

Utilizou-se um questionário sistematizado elaborado pelos pesquisadores com o objetivo de obter informações referentes às variáveis sociodemográficas (idade, sexo, escolaridade, estado civil, ocupação, renda e arranjo familiar) e clínicas (tempo de diagnóstico da doença e complicações crônicas). A idade foi analisada como variável contínua, expressa por média, desvio padrão e variância. O tempo de diagnóstico foi categorizado em quatro grupos: ≤1 ano, 1–5 anos, 6–10 anos e >10 anos.

Para a avaliação das atitudes dos participantes em relação ao DM2, foi aplicado o instrumento *Diabetes Attitude Questionnaire* (ATT-19), traduzido para a língua portuguesa e validado no Brasil^(17)^. Esse instrumento avalia a medida de ajustamento psicológico para DM e foi desenvolvido em resposta à necessidade de avaliar os aspectos psicológicos e emocionais relacionados à doença. Ele consiste em 19 itens, que incluem seis fatores: a) estresse associado ao DM; b) receptividade ao tratamento; c) confiança no tratamento; d) eficácia pessoal; e) percepção sobre a saúde; f) aceitação social. As questões 11, 15 e 18 começam com o escore reverso. Cada resposta é medida pela escala de Likert de cinco pontos (discordo totalmente [escore 1] até concordo totalmente [escore 5]). O valor total da taxa-escore varia de 19 a 95 pontos. Um escore menor que 70 pontos indica a atitude negativa sobre a doença^(17)^.

O ponto de corte <70 foi adotado conforme a recomendação do estudo de validade brasileira do questionário, que estabelece esse valor como indicativo de atitude negativa. Adicionalmente, procedeu-se à análise complementar do escore total do ATT-19 como variável contínua, a fim de explorar possíveis associações sem perda de informação decorrente da dicotomização.

### Análise e Tratamento de Dados

As análises estatísticas foram realizadas por meio do *software* RStudio (versão 2025.05.0), utilizando o pacote *dplyr* para a manipulação e a organização dos dados. As variáveis contínuas foram descritas por meio de medidas de tendência central e de dispersão, incluindo média, desvio padrão e variância. Para as variáveis categóricas, foram calculadas frequências absolutas e relativas, de modo a caracterizar a distribuição dos dados.

Para investigar os fatores associados à atitude negativa em relação ao DM2, foram ajustados modelos de regressão logística. Primeiramente, realizaram um modelo logístico simples para cada variável sociodemográfica para identificar preditores potenciais para o modelo múltiplo. Em seguida, todas as variáveis foram incluídas em um modelo inicial, verificando-se a presença de multicolinearidade por meio do Fator de Inflação da Variância, considerando-se valores >5 como indicativos de colinearidade elevada.

As variáveis foram mantidas no modelo com base em sua relevância teórica e epidemiológica, e sua exclusão foi analisada conjuntamente a partir de critérios de ajuste (Critério de Informação de Akaike [AIC]) e de colinearidade. O modelo 1 corresponde ao modelo ajustado que inclui todas as variáveis selecionadas com base em critérios teóricos e estatísticos. O modelo 2 refere-se ao modelo final, obtido após avaliação conjunta da significância, da colinearidade e da qualidade do ajuste.

Para cada variável, foram estimadas as Razões de Chance (*Odds Ratio* [OR]) com os respectivos Intervalos de Confiança de 95% (IC95%) e valor de p, adotando-se um nível de significância de p < 0,05. O teste de razão de verossimilhança (LRT) foi utilizado para avaliar a inclusão das variáveis preditoras no modelo múltiplo, em comparação com o modelo aninhado sem preditores, por meio do pacote *Imtest*.

O LRT foi utilizado para comparar o modelo aninhado, verificando se a inclusão conjunta das variáveis melhorava significativamente o ajuste em relação ao modelo reduzido. A comparação adicional entre os modelos considerou o AIC, adotando-se como mais adequado aquele com menor valor de AIC, indicando um melhor equilíbrio entre ajuste e parcimônia. Além disso, o teste de Hosmer-Lemeshow (HTL), com suporte do pacote *ResourceSelection*, foi aplicado para verificar a adequação do modelo, analisando a concordância entre as probabilidades previstas e observadas.

### Aspectos Éticos

O projeto de pesquisa foi aprovado pelo Comitê de Ética em Pesquisa da Universidade Estadual do Maranhão, sob o Parecer nº 5.967.691 e o Certificado de Apresentação para Apreciação Ética nº 67497823.0.0000.5554. Todos os entrevistados foram previamente informados dos objetivos e métodos da pesquisa, e mediante concordância em participar, assinaram o Termo de Consentimento Livre e Esclarecido.

## RESULTADOS

A amostra foi composta por 129 participantes com média de idade de 64,39 ± 11,89 anos, indicando o predomínio de indivíduos idosos. Houve uma maior prevalência do sexo feminino, representando 84 (65,12%) participantes. A maioria se autodeclarou parda, totalizando 63 (48,84%) indivíduos. Quanto ao estado civil, 64 (49,61%) estavam casados ou em união estável. Em relação à escolaridade, prevaleceu o ensino fundamental, com 49 (37,98%) indivíduos. A ocupação mais frequente foi a aposentadoria, relatada por 73 (56,59%) participantes. A maior parte dos entrevistados possuía renda de até um salário mínimo (79; 61,24%) e residia com familiares (52; 40,31%) (Tabela 1).

**Tabela 1:** Perfil sociodemográfico de participantes e atitude em relação à doença segundo o *Diabetes Attitude Questionnaire* – Caxias, MA, Brasil, 2025.

	Atitude negativa (n = 92)	Atitude positiva (n = 37)	Total N = 129
Idade				
Média	63,5	66,6	64,39
Desvio padrão	11	13,7	11,89
Variância	122	187,7	141,55
	**n (%)**	**n (%)**	**n (%)**
Sexo				
Feminino	57 (61,96)	27 (72,97)	84 (65,12)
Masculino	35 (38,04)	10 (27,03)	45 (34,88)
Etnia				
Parda	51 (55,43)	12 (32,43)	63 (48,84)
Preta	25 (27,17)	17 (45,95)	42 (32,56)
Branca	16 (17,39)	8 (21,62)	24 (18,60)
Estado civil				
Casado/união estável	46 (50,00)	18 (48,65)	64 (49,61)
Solteiro	27 (29,35)	10 (27,03)	37 (28,68)
Viúvo	17 (18,48)	8 (21,62)	25 (19,38)
Divorciado	2 (2,17)	1 (2,70)	3 (2,33)
Escolaridade				
Alfabetização	25 (27,17)	8 (21,62)	33 (25,58)
Ensino fundamental	38 (41,30)	11 (29,73)	49 (37,98)
Ensino médio	12 (13,04)	6 (16,22)	18 (13,95)
Ensino superior/especialização	5 (5,43)	4 (10,81)	9 (6,98)
Sem estudo	12 (13,04)	8 (21,62)	20 (15,50)
Ocupação				
Aposentado	52 (56,52)	21 (56,76)	73 (56,59)
Autônomo	31 (33,70)	14 (37,84)	45 (34,88)
Trabalhador assalariado	5 (5,43)	1 (2,70)	6 (4,65)
Outro	4 (4,35)	1 (2,70)	5 (3,88)
Renda				
Até um salário	57 (61,96)	22 (59,46)	79 (61,24)
Mais de um salário	6 (6,52)	3 (8,11)	9 (6,98)
Sem renda	29 (31,52)	12 (32,43)	41 (31,78)
Moradia				
Apenas com o cônjuge	15 (16,30)	2 (5,41)	17 (13,18)
Apenas familiares	36 (39,13)	16 (43,24)	52 (40,31)
Cônjuge e familiares	26 (28,26)	11 (29,73)	37 (28,68)
Mora sozinho	15 (16,30)	8 (21,62)	23 (17,83)

Fonte: os autores, 2025.

A Tabela 1 também demonstra os escores obtidos na avaliação das atitudes dos usuários com DM2 por meio do ATT-19. Verificou-se que a maioria dos participantes apresentou pontuação inferior a 70 pontos, indicativo de atitude negativa em relação à doença, totalizando 92 (71,32%) indivíduos.

Quanto às características clínicas, observou-se que 42 (32,56%) participantes conviviam com o diagnóstico de DM2 há um período de seis a dez anos. Em relação às complicações associadas à doença, predominou a ausência de alterações associadas à doença, totalizando 101 (78,29%) indivíduos. Entre aqueles que apresentaram complicações, a retinopatia foi a prevalente, totalizando 19 (14,73%) indivíduos (Tabela 2). As informações referentes às complicações clínicas foram obtidas por meio de consulta aos prontuários eletrônicos dos pacientes da unidade.

**Tabela 2:** Perfil clínico dos participantes segundo a atitude em relação à doença de acordo com o *Diabetes Attitude Questionnaire* – Caxias, MA, Brasil, 2025.

	Atitude negativa (n = 92)	Atitude positiva (n = 37)	Total N = 129
	n (%)	n (%)	n (%)
Tempo de diagnóstico (em anos)			
< 1	8 (8,70)	2 (5,41)	10 (7,75)
1 a 5	27 (29,35)	12 (32,43)	39 (30,23)
6 a 10	30 (32,61)	12 (32,43)	42 (32,56)
> 10	27 (29,35)	11 (29,73)	38 (29,46)
Complicações				
Não tem	74 (80,43)	27 (72,97)	101 (78,29)
Retinopatia	12 (13,04)	7 (18,92)	19 (14,73)
Renal	2 (2,17)	2 (5,41)	4 (3,10)
Vascular	2 (2,17)	1 (2,70)	3 (2,33)
Neuropatia	2 (2,17)	0 (0,00)	2 (1,55)

Fonte: os autores, 2025.

Os testes de associação (qui-quadrado e exato de Fisher) entre as variáveis sociodemográficas, clínicas e a atitude (negativa/positiva) não apresentaram significância estatística (p < 0,05). Diante disso, foi realizada uma análise de regressão logística para identificar os possíveis fatores associados à atitude negativa em relação ao DM2 (Tabela 3).

**Tabela 3:** Regressão logística múltipla: fatores associados à visão negativa das pessoas diagnosticadas com diabetes mellitus tipo 2 em relação à doença segundo o *Diabetes Attitude Questionnaire* – Caxias, MA, Brasil, 2025.

Variáveis inclusas no modelo	Modelo 1	Modelo 2
	OR (IC95%) Valor de p	OR (IC95%) Valor de p
Idade	0,95 (0,89; 1) 0,053	0,96 (0,92; 1,01) 0,095
Sexo (Ref = feminino)		
Masculino	2,96 (1,05; 9,27) 0,049*	2,66 (0,97; 8) 0,067
Etnia (Ref = branca)		
Parda	2,36 (0,69; 8,04) 0,166	2,36 (0,7; 8) 0,162
Preta	0,67 (0,18; 2,30) 0,530	0,66 (0,18; 2,23) 0,51
Estado civil (Ref = casado/união estável)			
Divorciado	2,37(0,1; 82,79) 0,592	2,7 (0,13; 87,29) 0,52
Solteiro	2,46 (0,57; 11,05) 0,229	2,76 (0,66; 12,08) 0,166
Viúvo	3,58 (0,67;19,72) 0,136	4,33 (0,86; 22,94) 0,078
Escolaridade (Ref = alfabetização)			
Ensino fundamental	0,88 (0,23; 3,31) 0,852	0,89 (0,24; 3,29) 0,867
Ensino médio	0,38 (0,07; 1,87) 0,230	0,36 (0,07; 1,73) 0,201
Ensino superior/especialização	0,17 (0,02; 1,27) 0,085	0,18 (0,03; 1,07) 0,059
Sem estudo	0,58 (0,15; 2,24) 0,429	0,54 (0,14; 2,06) 0,367
Arranjo familiar (Ref = apenas com o cônjuge)			
Apenas familiares	0,16 (0,02; 1,17) 0,088	0,15 (0,02; 1,03) 0,07
Cônjuge e familiares	0,29 (0,03; 1,56) 0,22	0,31 (0,04; 1,65) 0,202
Mora sozinho	0,11 (0,01; 0,80) 0,040*	0,1 (0,01; 0,73) 0,032*
Renda salarial (Ref = até um salário)			
Mais de um salário	0,72 (0,11; 5,59) 0,733	–
Sem renda	0,50 (0,13; 1,75) 0,279	–
**Critérios de avaliação dos modelos**			
AIC	169,32	164,12
VIF	<5	<5
LRT (Valor de p)	0,1553	0,1153
HLT (Valor de p)	0,213	0,589

Legenda: Ref – categoria de referência;

*Valor de p significativo com 95% de confiança; AIC – Critério de Avaliação de Akaike; VIF – Fator de Inflação da Variância; LRT – teste de razão de verossimilhança; HLT – teste de Hosmer-Lemeshow; ICP5% – Intervalo de Confiança de 95%.

Fonte: os autores, 2025.

No modelo 1, o sexo masculino apresentou associação significativa com a atitude negativa (OR = 2,96; IC95%: 1,05–9,27; p = 0,049), indicando maior probabilidade de os homens manifestarem visão desfavorável em relação à doença em comparação com as mulheres. Além disso, morar sozinho demonstrou um efeito protetor (OR = 0,11; IC95%: 0,01–0,80; p = 0,040), indicando que, em comparação ao grupo de referência (apenas com o cônjuge), indivíduos que moram sozinhos apresentaram menor chance de atitude negativa.

No modelo 2, após ajustes, morar sozinho manteve associação protetora (OR = 0,10; IC95%: 0,01–0,73; p = 0,032). O sexo masculino apresentou apenas tendência à associação com atitude negativa (OR = 2,66; IC95%: 0,97–8,10; p = 0,067), não alcançando significância estatística ao nível de 5%. Nenhuma outra variável apresentou associação estatisticamente significativa nos modelos finais. Os modelos demonstraram bom ajuste segundo o HTL (p = 0,213 no modelo 1 e p = 0,389 no modelo 2), além de valores satisfatórios do AIC (169,32 e 164,12, respectivamente) (Tabela 3).

## DISCUSSÃO

O uso do ATT-19 identificou que 71,32% dos participantes apresentaram atitudes negativas em relação ao DM2. Em análises multivariadas, o sexo masculino esteve associado a uma maior chance de atitudes negativas (OR = 2,96, IC95%: 1,05–9,27; p = 0,049), enquanto morar sozinho apresentou efeito protetor (OR = 0,11; IC95%: 0,01–0,80; p = 0,040). Fatores como etnia, baixa escolaridade, renda mensal de até um salário mínimo e tempo de diagnóstico entre seis e dez anos caracterizam o perfil da amostra, sendo variáveis descritas sem associação estatística.

O uso de instrumentos validados representa uma estratégia essencial para a prática em saúde, uma vez que possibilita a padronização da linguagem entre profissionais, favorecendo a comunicação interdisciplinar^(18)^. Além disso, constitui uma ferramenta para a avaliação das respostas terapêuticas, a comparação de dados ao longo do tempo e a compreensão de aspectos relacionados ao manejo de condições crônicas, como o DM2, permitindo uma análise mais consistente do comportamento e das atitudes em relação à doença^(19)^.

A variável “idade” apresentou associação parcialmente significativa com a atitude negativa em relação ao diagnóstico de DM2, conforme observado no modelo 1 (OR = 0,95; IC95%: 0,89–1,00; p = 0,053). Embora o valor de p esteja ligeiramente acima do ponto de corte convencional para a significância estatística (p < 0,05), o resultado sugere uma tendência de que o aumento da idade esteja associado a uma menor probabilidade de desenvolver uma visão negativa em relação à doença.

Esse achado pode ser interpretado por meio de fatores psicossociais e de vivências com a condição crônica. Por estarem mais frequentemente expostos a experiências relacionadas ao adoecimento crônico, indivíduos idosos podem desenvolver estratégias adaptativas mais eficazes, maior resiliência e uma aceitação mais realista do processo de viver com o DM2. Possivelmente, eles também tenham uma percepção mais madura diante das limitações impostas pela condição^(20)^.

Na amostra, predominou o sexo feminino, resultado semelhante ao de outro estudo^(21)^, que trabalhou com 936 pessoas com DM2 e identificou 63,4% de mulheres. Essa predominância pode estar relacionada ao comportamento de autocuidado mais frequente no sexo feminino, à maior preocupação com a saúde e à busca regular por serviços de prevenção e acompanhamento^(22)^. Além disso, pesquisas sugerem que as mulheres tendem a aderir mais aos cuidados e tratamentos do DM2, o que pode explicar a maior detecção da doença nesse grupo^(20, 21)^.

No presente estudo, identificou-se que o sexo masculino apresentou maior escore de atitude negativa em relação ao DM2, com associação estatisticamente significativa no modelo 1. Contudo, como no modelo 2 essa associação não manteve significância estatística (p = 0,067), o achado deve ser interpretado como tendência. Esse resultado está alinhado a evidências que apontam para um menor engajamento dos homens em práticas preventivas e uma menor adesão ao tratamento de doenças crônicas, quando comparados às mulheres^(23)^.

A maioria dos participantes se autodeclarou de etnia parda, o que pode estar relacionado às características étnicas da população brasileira e da região onde a pesquisa foi realizada. Estudos recentes apresentam achados semelhantes, reforçando o aumento da autodeclaração étnico-racial em levantamentos de saúde, permitindo que o indivíduo se identifique conforme sua própria percepção^(24, 25)^.

Esse fenômeno pode estar associado ao fortalecimento das políticas públicas voltadas à equidade em saúde. Compreender a distribuição étnico-racial entre pessoas com DM2 é essencial, uma vez que fatores socioeconômicos e culturais, bem como possíveis desigualdades de acesso aos serviços de saúde, podem impactar o manejo da doença e influenciar as atitudes em relação ao tratamento.

A baixa escolaridade foi predominante nesta pesquisa, podendo refletir as iniquidades históricas no acesso à educação até o século XX no Brasil. Achados semelhantes são descritos na literatura, como em um estudo que avaliou o conhecimento sobre diabetes, a atitude em relação ao autocuidado e fatores associados, indicando que a escolaridade pode estar associada a um menor conhecimento sobre a doença^(26)^. Nesse sentido, a literatura descreve que essa condição pode estar associada a dificuldades de compreensão e de seguimento das condutas terapêuticas, o que pode repercutir em maiores riscos à saúde.

De forma semelhante, um estudo recente identificou uma associação significativa entre baixo nível educacional e maior risco de DM2. Os autores destacam que a escolaridade constitui um importante determinante social da saúde, podendo influenciar o acesso às informações, aos serviços de saúde e às práticas de autocuidado relacionadas à prevenção e ao manejo da doença^(27)^.

Na amostra, predominaram participantes aposentados e com renda mensal de um salário mínimo, achado consistente com pesquisas anteriores^(21, 26)^. A baixa renda é um fator preocupante, pois pode representar uma barreira para a manutenção das necessidades básicas do indivíduo, além de dificultar o acesso contínuo a recursos necessários para o controle do DM. A renda familiar limitada permanece como um fator crítico no contexto do DM2, uma vez que condições de vida instáveis, a duração prolongada do tratamento e os custos relacionados ao cuidado da saúde podem representar desafios para o manejo da doença. Além disso, as restrições financeiras podem dificultar a adoção e a manutenção de hábitos alimentares saudáveis, componente fundamental para o controle glicêmico. Nesse contexto, a literatura descreve que fatores socioeconômicos desfavoráveis podem influenciar o acesso aos recursos necessários para o tratamento, repercutindo negativamente na gestão da condição de saúde e na qualidade de vida dos indivíduo^(28)^.

Em relação ao estado civil, observou-se uma maior proporção de participantes casados ou em união estável. Resultados semelhantes foram identificados em um estudo que analisou a correlação entre sintomas de depressão, atitude e autocuidado em idosos com DM2, realizado com 144 participantes da APS de João Pessoa^(29)^. O estado civil tem sido descrito na literatura como um fator relacionado à dinâmica familiar e a aspectos do autocuidado, uma vez que o arranjo familiar pode estar associado à adesão ao tratamento^(30)^.

Apesar da influência positiva atribuída ao suporte familiar, a análise multivariada deste estudo revelou que morar sozinho apresentou associação protetora em relação à atitude negativa em relação ao DM2 (OR = 0,11; IC95%: 0,01–0,80; p = 0,040). Considerando o número reduzido de participantes que moravam sozinhos e a amplitude dos intervalos de confiança, esse achado deve ser interpretado com cutela, não sendo possível descartar a instabilidade da estimativa ou a influência de fatores não mensurados^(28)^.

No presente estudo, observou-se que a maior parte dos participantes convivia com o diagnóstico de DM2 há um período de seis a dez anos, característica que reforça a natureza crônica e progressiva da doença. Ademais, 78,29% não apresentavam complicações relacionadas ao DM, enquanto, entre aqueles com alterações, a retinopatia foi a mais prevalente (14,73%). A literatura aponta que o tempo prolongado de diagnóstico está diretamente relacionado ao maior risco de complicações micro e macrovasculares, devido ao impacto cumulativo do descontrole glicêmico e da resistência à insulina ao longo dos anos^(3,5)^. Essas complicações, especialmente as que afetam a visão e as extremidades inferiores, estão associadas à redução da capacidade funcional e ao declínio da qualidade de vida, dificultando a adesão ao autocuidado^(5,6)^.

Entre as limitações do estudo, destaca-se o delineamento transversal, que impede estabelecer as relações temporais ou casais entre as variáveis. A amostra por conveniência pode ter introduzido o viés de seleção, limitando a generalização dos achados. O tamanho amostral reduzido em algumas categorias pode ter comprometido o poder estatístico. Embora as complicações tenham sido obtidas por prontuários, outras informações dependeram de autorrelato dos participantes, o que pode ter influências de interpretações pessoais dos participantes a fornecerem respostas socialmente aceitas.

Adicionalmente, a ausência de medidas objetivas de controle glicêmico, como a hemoglobina glicada, limita a avaliação mais precisa da condição clínica dos participantes. A falta de informações detalhadas sobre o tratamento, incluindo o uso de medicamentos, a adesão terapêutica e o tempo de acompanhamento, também restringe a compreensão dos fatores que podem influenciar as atitudes observadas. Soma-se a isso a possibilidade de confundimento residual, decorrente de variáveis não mensuradas ou controladas no modelo analítico.

Além disso, o perfil sociodemográfico da amostra, predominantemente composta por mulheres e baixa escolaridade, pode não refletir todas as realidades existentes entre indivíduos com DM2. Esses fatores devem ser considerados na interpretação dos achados e indicam a necessidade de futuras pesquisas que envolvam amostras mais diversificadas, a fim de ampliar a compreensão sobre as atitudes em distintos contextos sociais.

## CONCLUSÃO

Os achados deste estudo evidenciam a complexidade das atitudes de pessoas com DM2 em relação ao diagnóstico e ao tratamento da doença. Observou-se elevada frequência de atitudes negativas em relação ao DM2 entre os participantes avaliados pelo ATT-19. Entre as variáveis analisadas, o sexo masculino apresentou maior chance de atitude negativa, enquanto morar sozinho esteve associado a uma menor chance de atitude negativa. Esses achados contribuem para a compreensão das atitudes relacionadas ao DM entre os participantes investigados na APS.

## Data Availability

Todo conjunto de dados que dá suporte aos resultados deste estudo foi publicado no próprio artigo.

# Translation: Análisis de las actitudes de las personas con diabetes mellitus tipo 2 en la Atención Primaria de Salud

**DOI:** 10.1590/1980-220X-REEUSP-2025-0549es

**Authors:** Amilton Diniz dos Santos, Ravena Melo e Silva, Maria Rita Sousa da Silva, Irisdalva França Soares Brito, Maria Edileuza Soares Moura

**Affiliations:** 1 Universidade Estadual do Maranhão, Programa de Pós-graduação em Biodiversidade, Ambiente e Saúde, Caxias, MA, Brasil.; 2 Universidade Estadual do Maranhão, Departamento de Ciências da Saúde, Caxias, MA, Brasil.; 3 Universidade Estadual do Maranhão, Programa de Residência Multiprofissional em Saúde da Família, Caxias, MA, Brasil.

**Keywords:** Actitud, Atención Primaria de Salud, Autocuidado, Educación en Salud, Diabetes Mellitus Tipo 2

## Abstract

**Objetivo:**

Analizar las actitudes de las personas con diabetes mellitus tipo 2 respecto a la enfermedad.

**Método:**

Se trata de un estudio transversal realizado en la Atención Primaria de Salud con 129 participantes diagnosticados con diabetes mellitus tipo 2. Se aplicó el instrumento *Diabetes Attitude Questionnaire* (ATT-19). El análisis estadístico incluyó regresión logística, con verificación de multicolinealidad, ajuste del modelo y significación estadística.

**Resultados:**

Se observó un predominio de mujeres (n = 84; 65,12 %), personas que se identificaron como de raza mestiza (n = 63; 48,84 %), jubilados (n = 73; 56,59 %), con educación primaria (n = 49; 37,98 %) y casados o en unión estable (n = 64; 49,61 %). El sexo masculino presentó una tendencia a asociarse con una actitud negativa (OR = 2,66; IC 95 %: 0,97–8,10; p = 0,067), mientras que vivir solo demostró un efecto protector (OR = 0,10; IC 95 %: 0,01–0,73; p = 0,032).

**Conclusión:**

Se evidenció el predominio de actitudes negativas frente a la diabetes mellitus tipo 2, medidas mediante el ATT-19, con una mayor actitud negativa asociada al sexo masculino y un efecto protector para quienes viven solos, lo que indica la necesidad de estrategias enfocadas en las actitudes en la atención primaria de salud.

## INTRODUCCIÓN

Las enfermedades crónicas no transmisibles (ECNT) abarcan un grupo de enfermedades de evolución prolongada, generalmente de inicio insidioso y progresión lenta. Se caracterizan por su persistencia a lo largo del tiempo y por la necesidad de cuidados continuos orientados al control de los síntomas, la reducción de las complicaciones y el mantenimiento de la funcionalidad^(1)^. Entre las principales ECNT, se destacan las enfermedades cardiovasculares, los cánceres, las enfermedades respiratorias crónicas y la diabetes mellitus (DM). Estas afecciones afectan la calidad de vida de las personas afectadas, interfieren en su autonomía y en la realización de las actividades cotidianas, y requieren un seguimiento regular por parte de los profesionales de la salud^(2)^.

Entre las ECNT, la diabetes mellitus tipo 2 (DM2) es una de las afecciones crónicas más prevalentes y de mayor impacto para los sistemas de salud, y se caracteriza por una hiperglucemia persistente derivada de alteraciones en la producción o en la acción de la insulina^(3,6)^. A nivel mundial, la DM2 representa más del 90 % de todos los casos, y su prevalencia ha aumentado en varios países, impulsada por cambios demográficos, la urbanización y una mayor exposición a factores de riesgo relacionados con el comportamiento^(7)^. En Brasil se observa una tendencia similar, con un crecimiento significativo en el número de personas que viven con la enfermedad en las últimas décadas, lo que refuerza la necesidad de estrategias de atención enfocadas en el manejo de la afección^(8)^.

Ante este panorama, la lucha contra la DM2 está alineada con la meta 3.4 del Objetivo de Desarrollo Sostenible 3 (Salud y bienestar), que propone reducir en un 30 % la mortalidad prematura por enfermedades no transmisibles para 2030. Esta meta contempla intervenciones eficaces en el sistema de salud, incluyendo la prevención, el control de riesgos, el tratamiento adecuado y el acceso a la atención esencial^(9)^.

En este contexto, el control de la DM2 requiere un seguimiento clínico periódico y la participación activa del propio paciente en el manejo de la enfermedad. La adopción de prácticas de autocuidado, como la adherencia al plan alimenticio y farmacológico, la práctica de actividad física regular y el monitoreo de los signos y síntomas, es fundamental para alcanzar el equilibrio metabólico y reducir las complicaciones^(10)^. Sin embargo, la efectividad de estas medidas está directamente relacionada con el grado de adherencia al tratamiento, el cual se ve influenciado por diferentes factores, entre los que se destacan las actitudes y creencias que tiene la persona respecto a su estado de salud, las cuales son determinantes para mantener comportamientos saludables a lo largo del tiempo^(11)^.

La actitud puede entenderse como una disposición cognitiva que orienta la toma de decisiones ante las exigencias del tratamiento y el enfrentamiento a la enfermedad. Se trata de un componente subjetivo que refleja la forma en que la persona percibe, acepta y se posiciona respecto a su propio estado de salud^(12)^. Cuando es positiva, esta actitud favorece la participación en prácticas de autocuidado y la adherencia al tratamiento, lo que contribuye a mejores resultados clínicos y a una sensación de bienestar. Esta postura está fuertemente influenciada por el conocimiento que el individuo va adquiriendo con el tiempo, ya sea a través de experiencias personales o de orientaciones profesionales, que le brindan apoyo para el manejo de su enfermedad^(13,14,15)^.

A pesar de la relevancia de este constructo, aún son limitadas las investigaciones que analizan las actitudes de las personas con DM2 en el contexto de la Atención Primaria de Salud (APS), particularmente en regiones del noreste de Brasil. La mayor parte de la investigación se concentra en indicadores clínicos, prestándose menos atención a las dimensiones relacionadas con las actitudes frente a la enfermedad.

En este sentido, investigar las actitudes de las personas con DM2 atendidas en la APS puede contribuir a ampliar la comprensión sobre cómo estos individuos perciben y se posicionan frente a la enfermedad en su contexto de atención. Este conocimiento puede aportar información para la planificación de acciones de salud alineadas con las experiencias y percepciones de los usuarios. De esta manera, el presente estudio tuvo como objetivo analizar las actitudes de las personas con DM2 en relación con la enfermedad.

## MÉTODO

### Tipo de Estudio

Se trata de un estudio transversal de enfoque cuantitativo y carácter exploratorio realizado en el municipio de Caxias, ubicado en el estado de Maranhão. El diseño y el informe de la investigación siguieron las recomendaciones de la *lista de verificación STrengthening the Reporting of OBservational studies in Epidemiology*, dirigida a estudios transversales.

### Lugar del Estudio

Se seleccionó a la población atendida por el equipo de atención primaria de salud (APS) de la zona urbana del municipio. La selección de la Unidad Básica de Salud se justifica por su carácter de unidad modelo, además de ser un campo de práctica para los profesionales vinculados al Programa de Residencia Multiprofesional en Salud Familiar de la Universidad Estatal de Maranhão. Cabe destacar, además, su amplia área de cobertura y el número significativo de usuarios con diagnóstico de DM2, lo que la convierte en un escenario prometedor para el desarrollo del estudio.

### Población y Criterios de Selección

El equipo de atención primaria de salud (APS) atiende a 198 personas con diagnóstico de DM2. Se incluyeron en el estudio personas con diagnóstico clínico de DM2, de 18 años o más, registradas y atendidas por el equipo de APS seleccionado, y con capacidad cognitiva intacta para responder al instrumento de Recolección de datos, aunque con la ayuda de un familiar, cuidador o acompañante. Se excluyó a las personas con afecciones que dificultaran la comunicación verbal y/o la comprensión del instrumento aplicado.

La muestra se seleccionó por conveniencia, de acuerdo con la accesibilidad y la disponibilidad de los participantes durante el período de Recolección de datos, lo que puede introducir un sesgo de selección y limitar la representatividad de la población atendida por el equipo y la generalización de los hallazgos. El diagnóstico de DM2 se confirmó mediante el registro en el expediente médico electrónico del centro de salud, tomando en cuenta los diagnósticos clínicos previamente establecidos por un profesional médico, de acuerdo con el seguimiento en la APS.

### Cálculo del Tamaño de la Muestra

El cálculo de la muestra se realizó utilizando la siguiente fórmula para poblaciones finitas: n = N·Z^2^·p·(1–p)/(N–1)·e^2^ + Z^2^·p·(1–p)^(16)^. Se consideró una población heterogénea, un nivel de confianza del 95 % (Z = 1,96), un margen de error del 5 % (e = 0,05) y una prevalencia estimada del 50 % (p = 0,5), lo que dio como resultado una muestra de 131 participantes. Durante la etapa de recolección de datos, se excluyó a dos participantes de la investigación por presentar limitaciones que dificultaban la comunicación verbal y la comprensión del instrumento aplicado, lo que dio como resultado una muestra final de 129 participantes.

### Recolección de Datos

La recolección de datos se llevó a cabo entre marzo y junio de 2025 en el centro de salud y en el domicilio de los participantes, según la disponibilidad de estos.

Se contó con el apoyo de agentes comunitarios de salud, encargados del seguimiento de los participantes. La aplicación de los cuestionarios se llevó a cabo de manera individual, en un lugar privado, con el fin de garantizar la privacidad, evitar situaciones incómodas y asegurar la confidencialidad de la información. La duración promedio de cada entrevista fue de aproximadamente 25 minutos (DE = 5), con un tiempo mínimo de 20 minutos y un máximo de 40 minutos.

Se utilizó un cuestionario sistematizado elaborado por los investigadores con el objetivo de obtener información sobre las variables sociodemográficas (edad, sexo, nivel educativo, estado civil, ocupación, ingresos y estructura familiar) y clínicas (tiempo transcurrido desde el diagnóstico de la enfermedad y complicaciones crónicas). La edad se analizó como variable continua, expresada mediante la media, la desviación estándar y la varianza. El tiempo transcurrido desde el diagnóstico se clasificó en cuatro grupos: ≤1 año, 1–5 años, 6–10 años y >10 años.

Para evaluar las actitudes de los participantes respecto a la DM2, se aplicó el instrumento *Diabetes Attitude Questionnaire* (ATT-19), traducido al portugués y validado en Brasil^(17)^. Este instrumento evalúa el grado de adaptación psicológica a la DM y se desarrolló en respuesta a la necesidad de evaluar los aspectos psicológicos y emocionales relacionados con la enfermedad. Consta de 19 ítems, que incluyen seis factores: a) estrés asociado a la DM; b) receptividad al tratamiento; c) confianza en el tratamiento; d) eficacia personal; e) percepción de la salud; f) aceptación social. Las preguntas 11, 15 y 18 comienzan con puntuación inversa. Cada respuesta se mide mediante una escala de Likert de cinco puntos (desde «Totalmente en desacuerdo» [puntuación 1] hasta «Totalmente de acuerdo» [puntuación 5]). El valor total de la puntuación oscila entre 19 y 95 puntos. Una puntuación inferior a 70 puntos indica una actitud negativa hacia la enfermedad^(17)^.

Se adoptó el punto de corte <70 de acuerdo con la recomendación del estudio de validez del cuestionario en Brasil, que establece este valor como indicativo de una actitud negativa. Además, se llevó a cabo un análisis complementario de la puntuación total del ATT-19 como variable continua, con el fin de explorar posibles asociaciones sin pérdida de información derivada de la dicotomización.

### Análisis y Procesamiento de Datos

Los análisis estadísticos se realizaron mediante el *software* RStudio (versión 2025.05.0), utilizando el paquete *dplyr* para la manipulación y organización de los datos. Las variables continuas se describieron mediante medidas de tendencia central y dispersión, incluyendo la media, la desviación estándar y la varianza. Para las variables categóricas, se calcularon las frecuencias absolutas y relativas, con el fin de caracterizar la distribución de los datos.

Para investigar los factores asociados a la actitud negativa hacia la DM2, se ajustaron modelos de regresión logística. En primer lugar, se realizó un modelo logístico simple para cada variable sociodemográfica con el fin de identificar posibles predictores para el modelo múltiple. A continuación, se incluyeron todas las variables en un modelo inicial, verificando la presencia de multicolinealidad mediante el factor de inflación de la varianza, considerando que los valores >5 son indicativos de alta colinealidad.

Las variables se mantuvieron en el modelo en función de su relevancia teórica y epidemiológica, y su exclusión se analizó de manera conjunta a partir de criterios de ajuste (Criterio de Información de Akaike [AIC]) y de colinealidad. El modelo 1 corresponde al modelo ajustado que incluye todas las variables seleccionadas con base en criterios teóricos y estadísticos. El modelo 2 se refiere al modelo final, obtenido tras una evaluación conjunta de la significación, la colinealidad y la calidad del ajuste.

Para cada variable, se estimaron las razones de probabilidad (*Odds Ratio* [OR]) con sus respectivos intervalos de confianza del 95 % (IC95 %) y valor de p, adoptando un nivel de significancia de p < 0,05. Se utilizó la prueba de la razón de verosimilitud (LRT) para evaluar la inclusión de las variables predictoras en el modelo múltiple, en comparación con el modelo anidado sin predictores, mediante el paquete *Imtest*.

La LRT se utilizó para comparar el modelo anidado, verificando si la inclusión conjunta de las variables mejoraba significativamente el ajuste en relación con el modelo reducido. La comparación adicional entre los modelos tomó en cuenta el AIC, adoptándose como más adecuado aquel con el valor de AIC más bajo, lo que indica un mejor equilibrio entre ajuste y parsimonia. Además, se aplicó la prueba de Hosmer-Lemeshow (HTL), con el apoyo del paquete *ResourceSelection*, para verificar la adecuación del modelo, analizando la concordancia entre las probabilidades predichas y observadas.

### Aspectos Éticos

El proyecto de investigación fue aprobado por el Comité de Ética en Investigación de la Universidad Estatal de Maranhão, mediante el Dictamen n.º 5.967.691 y el Certificado de Presentación para Evaluación Ética n.º 67497823.0.0000.5554. Todos los entrevistados fueron informados previamente sobre los objetivos y métodos de la investigación y, tras aceptar participar, firmaron el formulario de consentimiento libre e informado.

## RESULTADOS

La muestra estuvo compuesta por 129 participantes con una edad promedio de 64,39 ± 11,89 años, lo que indica un predominio de personas de la tercera edad. Hubo una mayor prevalencia del sexo femenino, que representó a 84 (65,12 %) participantes. La mayoría se autodefinió como de raza mestiza, lo que sumó un total de 63 (48,84 %) personas. En cuanto al estado civil, 64 (49,61 %) estaban casados o en unión estable. En cuanto al nivel educativo, predominó la educación primaria, con 49 (37,98 %) personas. La ocupación más frecuente fue la jubilación, declarada por 73 (56,59 %) participantes. La mayor parte de los entrevistados tenía un ingreso de hasta un salario mínimo (79; 61,24 %) y residía con familiares (52; 40,31 %) (Tabla 1).

**Tabla 1:** Perfil sociodemográfico de los participantes y actitud frente a la enfermedad según el *Diabetes Attitude Questionnaire* – Caxias, MA, Brasil, 2025.

	Actitud negativa (n = 92)	Actitud positiva (n = 37)	Total N = 129
Edad			
Media	63,5	66,6	64,39
Desviación estándar	11	13,7	11,89
Varianza	122	187,7	141,55
	**n (%)**	**n (%)**	**n (%)**
Sexo			
Mujer	57 (61,96)	27 (72,97)	84 (65,12)
Masculino	35 (38,04)	10 (27,03)	45 (34,88)
Origen étnico			
Mestizo	51 (55,43)	12 (32,43)	63 (48,84)
Negra	25 (27,17)	17 (45,95)	42 (32,56)
Blanca	16 (17,39)	8 (21,62)	24 (18,60)
Estado civil			
Casado/en pareja de hecho	46 (50,00)	18 (48,65)	64 (49,61)
Soltero	27 (29,35)	10 (27,03)	37 (28,68)
Viudo	17 (18,48)	8 (21,62)	25 (19,38)
Divorciado	2 (2,17)	1 (2,70)	3 (2,33)
Nivel educativo			
Alfabetización	25 (27,17)	8 (21,62)	33 (25,58)
Educación primaria	38 (41,30)	11 (29,73)	49 (37,98)
Educación secundaria	12 (13,04)	6 (16,22)	18 (13,95)
Educación superior/especialización	5 (5,43)	4 (10,81)	9 (6,98)
Sin estudios	12 (13,04)	8 (21,62)	20 (15,50)
Ocupación			
Jubilado	52 (56,52)	21 (56,76)	73 (56,59)
Autónomo	31 (33,70)	14 (37,84)	45 (34,88)
Trabajador por cuenta ajena	5 (5,43)	1 (2,70)	6 (4,65)
Otros	4 (4,35)	1 (2,70)	5 (3,88)
Ingresos			
Hasta un salario	57 (61,96)	22 (59,46)	79 (61,24)
Más de un salario	6 (6,52)	3 (8,11)	9 (6,98)
Sin ingresos	29 (31,52)	12 (32,43)	41 (31,78)
Vivienda unifamiliar			
Solo con el cónyuge	15 (16,30)	2 (5,41)	17 (13,18)
Solo familiares	36 (39,13)	16 (43,24)	52 (40,31)
Cónyuge y familiares	26 (28,26)	11 (29,73)	37 (28,68)
Vive solo	15 (16,30)	8 (21,62)	23 (17,83)

Fuente: los autores, 2025.

La Tabla 1 también muestra las puntuaciones obtenidas en la evaluación de las actitudes de los usuarios con DM2 mediante el ATT-19. Se observó que la mayoría de los participantes obtuvieron una puntuación inferior a 70 puntos, lo que indica una actitud negativa hacia la enfermedad, con un total de 92 (71,32 %) personas.

En cuanto a las características clínicas, se observó que 42 (32,56 %) participantes llevaban entre seis y diez años conviviendo con el diagnóstico de DM2. En relación con las complicaciones asociadas a la enfermedad, predominó la ausencia de alteraciones relacionadas con la misma, con un total de 101 (78,29 %) individuos. Entre aquellos que presentaban complicaciones, la retinopatía fue la más frecuente, con un total de 19 (14,73 %) personas (Tabla 2). La información relativa a las complicaciones clínicas se obtuvo mediante la consulta de los historiales clínicos electrónicos de los pacientes del centro.

**Tabla 2:** Perfil clínico de los participantes según su actitud frente a la enfermedad, según el *Diabetes Attitude Questionnaire* – Caxias, MA, Brasil, 2025.

	Actitud negativa (n = 92)	Actitud positiva (n = 37)	Total N = 129
	n (%)	n (%)	n (%)
Tiempo transcurrido desde el diagnóstico (en años)			
< 1	8 (8,70)	2 (5,41)	10 (7,75)
1 a 5	27 (29,35)	12 (32,43)	39 (30,23)
6 a 10	30 (32,61)	12 (32,43)	42 (32,56)
> 10	27 (29,35)	11 (29,73)	38 (29,46)
Complicaciones			
Ninguna	74 (80,43)	27 (72,97)	101 (78,29)
Retinopatía	12 (13,04)	7 (18,92)	19 (14,73)
Renal	2 (2,17)	2 (5,41)	4 (3,10)
Vascular	2 (2,17)	1 (2,70)	3 (2,33)
Neuropatía	2 (2,17)	0 (0,00)	2 (1,55)

Fuente: los autores, 2025.

Las pruebas de asociación (chi-cuadrado y exacta de Fisher) entre las variables sociodemográficas, clínicas y la actitud (negativa/positiva) no mostraron significación estadística (p < 0,05). Ante ello, se llevó a cabo un análisis de regresión logística para identificar los posibles factores asociados a la actitud negativa respecto a la DM2 (Tabla 3).

**Tabla 3:** Regresión logística múltiple: factores asociados a la visión negativa de las personas diagnosticadas con diabetes mellitus tipo 2 respecto a la enfermedad según el *Diabetes Attitude Questionnaire* – Caxias, MA, Brasil, 2025.

Variables incluidas en el modelo	Modelo 1	Modelo 2
	OR (IC del 95 %) Valor de p	OR (IC del 95 %) Valor de p
Edad	0,95 (0,89; 1) 0,053	0,96 (0,92; 1,01) 0,095
Sexo (Ref. = femenino)		
Masculino	2,96 (1,05; 9,27) 0,049*	2,66 (0,97; 8) 0,067
Origen étnico (Ref. = blanco)		
Mestiza	2,36 (0,69; 8,04) 0,166	2,36 (0,7; 8) 0,162
Negra	0,67 (0,18; 2,30) 0,530	0,66 (0,18; 2,23) 0,51
Estado civil (Ref = casado/unión de hecho)		
Divorciado	2,37 (0,1; 82,79) 0,592	2,7 (0,13; 87,29) 0,52
Soltero	2,46 (0,57; 11,05) 0,229	2,76 (0,66; 12,08) 0,166
Viudo	3,58 (0,67; 19,72) 0,136	4,33 (0,86; 22,94) 0,078
Nivel educativo (Ref. = alfabetización)		
Educación primaria	0,88 (0,23; 3,31) 0,852	0,89 (0,24; 3,29) 0,867
Educación secundaria	0,38 (0,07; 1,87) 0,230	0,36 (0,07; 1,73) 0,201
Educación superior/especialización	0,17 (0,02; 1,27) 0,085	0,18 (0,03; 1,07) 0,059
Sin estudios	0,58 (0,15; 2,24) 0,429	0,54 (0,14; 2,06) 0,367
Convivencia familiar (Ref. = solo con el cónyuge)		
Solo familiares	0,16 (0,02; 1,17) 0,088	0,15 (0,02; 1,03) 0,07
Cónyuge y familiares	0,29 (0,03; 1,56) 0,22	0,31 (0,04; 1,65) 0,202
Vive solo	0,11 (0,01; 0,80) 0,040*	0,1 (0,01; 0,73) 0,032*
Ingresos salariales (Ref = hasta un salario)		
Más de un salario	0,72 (0,11; 5,59) 0,733	–
Sin ingresos	0,50 (0,13; 1,75) 0,279	–
**Criterios de evaluación de los modelos**		
AIC	169,32	164,12
VIF	<5	<5
LRT (valor p)	0,1553	0,1153
HLT (valor p)	0,213	0,589

Abreviaturas: Ref: categoría de referencia;

*Valor de p significativo con un nivel de confianza del 95 %; AIC: criterio de evaluación de Akaike; VIF: factor de inflación de la varianza; LRT: prueba de la razón de verosimilitud; HLT: prueba de Hosmer-Lemeshow; ICP5 %: intervalo de confianza del 95 %.

Fuente: los autores, 2025.

En el modelo 1, el sexo masculino presentó una asociación significativa con la actitud negativa (OR = 2,96; IC 95 %: 1,05–9,27; p = 0,049), lo que indica una mayor probabilidad de que los hombres manifiesten una visión desfavorable respecto a la enfermedad en comparación con las mujeres. Además, vivir solo mostró un efecto protector (OR = 0,11; IC 95 %: 0,01–0,80; p = 0,040), lo que indica que, en comparación con el grupo de referencia (solo con la pareja), las personas que viven solas presentaron una menor probabilidad de tener una actitud negativa.

En el modelo 2, tras los ajustes, vivir solo mantuvo una asociación protectora (OR = 0,10; IC 95 %: 0,01–0,73; p = 0,032). El sexo masculino presentó solo una tendencia a la asociación con una actitud negativa (OR = 2,66; IC 95 %: 0,97–8,10; p = 0,067), sin alcanzar significación estadística al nivel del 5 %. Ninguna otra variable presentó una asociación estadísticamente significativa en los modelos finales. Los modelos mostraron un buen ajuste según el HTL (p = 0,213 en el modelo 1 y p = 0,389 en el modelo 2), además de valores satisfactorios del AIC (169,32 y 164,12, respectivamente) (Tabla 3).

## DISCUSIÓN

El uso del ATT-19 reveló que el 71,32 % de los participantes presentaba actitudes negativas hacia la DM2. En los análisis multivariados, el sexo masculino se asoció con una mayor probabilidad de actitudes negativas (OR = 2,96; IC del 95 %: 1,05–9,27; p = 0,049), mientras que vivir solo tuvo un efecto protector (OR = 0,11; IC 95 %: 0,01–0,80; p = 0,040). Factores como el origen étnico, el bajo nivel educativo, los ingresos mensuales de hasta un salario mínimo y el tiempo transcurrido desde el diagnóstico de entre seis y diez años caracterizan el perfil de la muestra, siendo variables descritas sin asociación estadística.

El uso de instrumentos validados representa una estrategia esencial para la práctica en salud, ya que permite la estandarización del lenguaje entre profesionales, lo que favorece la comunicación interdisciplinaria^(18)^. Además, constituye una herramienta para la evaluación de las respuestas terapéuticas, la comparación de datos a lo largo del tiempo y la comprensión de aspectos relacionados con el manejo de afecciones crónicas, como la DM2, lo que permite un análisis más consistente del comportamiento y las actitudes frente a la enfermedad^(19)^.

La variable «edad» presentó una asociación parcialmente significativa con la actitud negativa respecto al diagnóstico de DM2, tal como se observó en el modelo 1 (OR = 0,95; IC 95 %: 0,89–1,00; p = 0,053). Aunque el valor de p se encuentra ligeramente por encima del umbral convencional de significación estadística (p < 0,05), el resultado sugiere una tendencia a que el aumento de la edad se asocie con una menor probabilidad de desarrollar una visión negativa de la enfermedad.

Este hallazgo puede interpretarse a través de factores psicosociales y de las experiencias vividas con la enfermedad crónica. Al estar expuestos con mayor frecuencia a experiencias relacionadas con la enfermedad crónica, las personas de edad avanzada pueden desarrollar estrategias de adaptación más eficaces, una mayor resiliencia y una aceptación más realista del proceso de vivir con la DM2. Es posible que también tengan una percepción más madura ante las limitaciones que impone la afección^(20)^.

En la muestra predominó el sexo femenino, un resultado similar al de otro estudio^(21)^, que trabajó con 936 personas con DM2 e identificó un 63,4 % de mujeres. Este predominio puede estar relacionado con un comportamiento de autocuidado más frecuente en las mujeres, una mayor preocupación por la salud y la búsqueda regular de servicios de prevención y seguimiento^(22)^. Además, las investigaciones sugieren que las mujeres tienden a adherirse más a los cuidados y tratamientos de la DM2, lo que puede explicar la mayor detección de la enfermedad en este grupo^(20,21)^.

En el presente estudio, se identificó que el sexo masculino presentó una puntuación más alta de actitud negativa hacia la DM2, con una asociación estadísticamente significativa en el modelo 1. Sin embargo, dado que en el modelo 2 esta asociación no mantuvo significación estadística (p = 0,067), el hallazgo debe interpretarse como una tendencia. Este resultado concuerda con la evidencia que apunta a una menor participación de los hombres en prácticas preventivas y una menor adherencia al tratamiento de enfermedades crónicas, en comparación con las mujeres^(23)^.

La mayoría de los participantes se autodeclararon de etnia parda, lo que puede estar relacionado con las características étnicas de la población brasileña y de la región donde se llevó a cabo la investigación. Estudios recientes presentan hallazgos similares, lo que refuerza el aumento de la autodeclaración étnico-racial en encuestas de salud, permitiendo que el individuo se identifique según su propia percepción^(24,25)^.

Este fenómeno puede estar asociado al fortalecimiento de las políticas públicas orientadas a la equidad en salud. Comprender la distribución étnico-racial entre las personas con DM2 es esencial, ya que los factores socioeconómicos y culturales, así como las posibles desigualdades en el acceso a los servicios de salud, pueden afectar el manejo de la enfermedad e influir en las actitudes respecto al tratamiento.

El bajo nivel educativo fue predominante en esta investigación, lo que podría reflejar las desigualdades históricas en el acceso a la educación hasta el siglo XX en Brasil. En la literatura se describen hallazgos similares, como en un estudio que evaluó el conocimiento sobre la diabetes, la actitud respecto al autocuidado y los factores asociados, indicando que el nivel educativo puede estar relacionado con un menor conocimiento sobre la enfermedad^(26)^. En este sentido, la literatura describe que esta condición puede estar relacionada con dificultades para comprender y seguir las pautas terapéuticas, lo que puede traducirse en mayores riesgos para la salud.

De manera similar, un estudio reciente identificó una asociación significativa entre un bajo nivel educativo y un mayor riesgo de DM2. Los autores destacan que el nivel educativo constituye un importante determinante social de la salud, ya que puede influir en el acceso a la información, a los servicios de salud y a las prácticas de autocuidado relacionadas con la prevención y el manejo de la enfermedad^(27)^.

En la muestra predominaron los participantes jubilados y con un ingreso mensual equivalente a un salario mínimo, un hallazgo consistente con investigaciones anteriores^(21,26)^.

Los bajos ingresos son un factor preocupante, ya que pueden representar un obstáculo para satisfacer las necesidades básicas de la persona, además de dificultar el acceso continuo a los recursos necesarios para el control de la DM. Los ingresos familiares limitados siguen siendo un factor crítico en el contexto de la DM2, ya que las condiciones de vida inestables, la duración prolongada del tratamiento y los costos relacionados con la atención médica pueden representar desafíos para el manejo de la enfermedad. Además, las restricciones financieras pueden dificultar la adopción y el mantenimiento de hábitos alimenticios saludables, un componente fundamental para el control glicémico. En este contexto, la literatura describe que los factores socioeconómicos desfavorables pueden influir en el acceso a los recursos necesarios para el tratamiento, lo que repercute negativamente en el manejo de la afección y en la calidad de vida de las personas^(28)^.

En cuanto al estado civil, se observó una mayor proporción de participantes casados o en unión estable. Se identificaron resultados similares en un estudio que analizó la correlación entre los síntomas de depresión, la actitud y el autocuidado en adultos mayores con DM2, realizado con 144 participantes de la APS de João Pessoa^(29)^. El estado civil se ha descrito en la literatura como un factor relacionado con la dinámica familiar y con aspectos del autocuidado, ya que la estructura familiar puede estar asociada a la adherencia al tratamiento^(30)^.

A pesar de la influencia positiva que se le atribuye al apoyo familiar, el análisis multivariante de este estudio reveló que vivir solo presentó una asociación protectora frente a la actitud negativa hacia la DM2 (OR = 0,11; IC 95 %: 0,01-0,80; p = 0,040). Teniendo en cuenta el reducido número de participantes que vivían solos y la amplitud de los intervalos de confianza, este hallazgo debe interpretarse con cautela, ya que no es posible descartar la inestabilidad de la estimación ni la influencia de factores no medidos^(28)^.

En el presente estudio, se observó que la mayoría de los participantes convivían con el diagnóstico de DM2 desde hacía un período de seis a diez años, característica que refuerza la naturaleza crónica y progresiva de la enfermedad. Además, el 78,29 % no presentaba complicaciones relacionadas con la DM, mientras que, entre aquellos con alteraciones, la retinopatía fue la más prevalente (14,73 %). La literatura señala que un tiempo prolongado desde el diagnóstico está directamente relacionado con un mayor riesgo de complicaciones micro y macrovasculares, debido al impacto acumulativo del descontrol glicémico y de la resistencia a la insulina a lo largo de los años^(3,5)^. Estas complicaciones, especialmente las que afectan la visión y las extremidades inferiores, están asociadas con la reducción de la capacidad funcional y el deterioro de la calidad de vida, lo que dificulta la adherencia al autocuidado^(5,6)^.

Entre las limitaciones del estudio, se destaca el diseño transversal, que impide establecer relaciones temporales o causales entre las variables. La muestra por conveniencia pudo haber introducido un sesgo de selección, lo que limita la generalización de los hallazgos. El tamaño reducido de la muestra en algunas categorías pudo haber comprometido el poder estadístico.

Aunque los datos sobre las complicaciones se obtuvieron de los expedientes médicos, otra información dependió del autoinforme de los participantes, lo que puede haber estado influido por interpretaciones personales de los participantes al proporcionar respuestas socialmente aceptadas.

Además, la ausencia de medidas objetivas del control glicémico, como la hemoglobina glicosilada, limita una evaluación más precisa del estado clínico de los participantes. La falta de información detallada sobre el tratamiento, incluyendo el uso de medicamentos, la adherencia terapéutica y el tiempo de seguimiento, también limita la comprensión de los factores que pueden influir en las actitudes observadas. A esto se suma la posibilidad de un sesgo residual, derivado de variables no medidas o no controladas en el modelo analítico.

Además, el perfil sociodemográfico de la muestra, compuesta predominantemente por mujeres y con bajo nivel educativo, podría no reflejar todas las realidades existentes entre las personas con DM2. Estos factores deben tenerse en cuenta al interpretar los hallazgos e indican la necesidad de futuras investigaciones que incluyan muestras más diversificadas, con el fin de ampliar la comprensión sobre las actitudes en distintos contextos sociales.

## CONCLUSIÓN

Los resultados de este estudio evidencian la complejidad de las actitudes de las personas con DM2 respecto al diagnóstico y al tratamiento de la enfermedad. Se observó una alta frecuencia de actitudes negativas hacia la DM2 entre los participantes evaluados mediante el ATT-19. Entre las variables analizadas, el sexo masculino presentó una mayor probabilidad de actitud negativa, mientras que vivir solo se asoció con una menor probabilidad de actitud negativa. Estos hallazgos contribuyen a comprender las actitudes relacionadas con la DM entre los participantes investigados en la APS.

## Data Availability

Todo el conjunto de datos que respalda los resultados de este estudio se publicó en el propio artículo.
